# Purinergic Signaling as a Regulator of Th17 Cell Plasticity

**DOI:** 10.1371/journal.pone.0157889

**Published:** 2016-06-20

**Authors:** Dominique Fernández, Felipe Flores-Santibáñez, Jocelyn Neira, Francisco Osorio-Barrios, Gabriela Tejón, Sarah Nuñez, Yessia Hidalgo, Maria Jose Fuenzalida, Daniel Meza, Gonzalo Ureta, Alvaro Lladser, Rodrigo Pacheco, Claudio Acuña-Castillo, Victoria Guixé, Francisco J. Quintana, Maria Rosa Bono, Mario Rosemblatt, Daniela Sauma

**Affiliations:** 1 Departamento de Biologia, Facultad de Ciencias, Universidad de Chile, Santiago, Chile; 2 Facultad de Ciencias Biologicas, Universidad Andres Bello, Santiago, Chile; 3 Fundacion Ciencia & Vida, Santiago, Chile; 4 Departamento de Biologia y Centro de Biotecnologia Acuicola (CBA), Facultad de Quimica y Biologia, Universidad de Santiago de Chile, Santiago, Chile; 5 Center for Neurologic Diseases, Brigham and Women’s Hospital, Harvard Medical School, Boston, Massachusetts, United States of America; INSERM, FRANCE

## Abstract

T helper type 17 (Th17) lymphocytes, characterized by the production of interleukin-17 and other pro-inflammatory cytokines, are present in intestinal lamina propria and have been described as important players driving intestinal inflammation. Recent evidence, supporting the notion of a functional and phenotypic instability of Th17 cells, has shown that Th17 differentiate into type 1 regulatory (Tr1) T cells during the resolution of intestinal inflammation. Moreover, it has been suggested that the expression of CD39 ectonucleotidase endows Th17 cells with immunosuppressive properties. However, the exact role of CD39 ectonucleotidase in Th17 cells has not been studied in the context of intestinal inflammation. Here we show that Th17 cells expressing CD39 ectonucleotidase can hydrolyze ATP and survive to ATP-induced cell death. Moreover, *in vitro*-generated Th17 cells expressing the CD39 ectonucleotidase produce IL-10 and are less pathogenic than CD39 negative Th17 cells in a model of experimental colitis in Rag^-/-^ mice. Remarkably, we show that CD39 activity regulates the conversion of Th17 cells to IL-10-producing cells *in vitro*, which is abrogated in the presence of ATP and the CD39-specific inhibitor ARL67156. All these data suggest that CD39 expression by Th17 cells allows the depletion of ATP and is crucial for IL-10 production and survival during the resolution of intestinal inflammation.

## Introduction

T helper 17 (Th17) cells play a fundamental protective role against infections caused by fungi and extracellular bacteria [1,2]. Th17 cells have been associated with inflammation and the pathogenesis of several autoimmune diseases in mice and humans [1], such as experimental autoimmune encephalomyelitis (EAE) [3], experimental colitis [4,5] and human inflammatory bowel disease (IBD) [6–10].

Th17 cells are endowed with remarkable functional plasticity, being able to differentiate into Th1 cells in lymphopenic hosts, during EAE and in antitumor immune responses [11–15]. Some groups have also reported the presence of IFN- γ -producing Th17 cells in humans and patients with Crohn’s disease [16,17]. Th17 cells can also differentiate into IL-10-producing cells during the resolution of inflammation [18–20]. Interestingly, using fate mapping mouse models, Flavell and colleagues have recently demonstrated that in the course of an inflammatory immune response, Th17 cells can transdifferentiate into type 1 regulatory T (Tr1) cells [21].

Two distinct types of Th17 cells have been defined: pathogenic or encephalitogenic Th17 cells and the so-called non-pathogenic Th17 cells. Some reports have demonstrated that Th17 cells generated with TGF-β1 and IL-6 are not pathogenic in the setting of EAE [18], whereas Th17 cells produced with IL-23 and without TGF-β1 are highly pathogenic in the same model [22–24]. Depending on the experimental setting, different cytokines produced by Th17 cells seem to drive their effector function. For instance, in tumor immunity, the production of IFN-γ by Th17 cells has proved to be determining the potential of Th17 cells to eradicate an established tumor [14]. Other studies revealed that the encephalitogenic properties of Th17 cells depend on GM-CSF production [23,25], whereas IL-10 production has been strongly related to non-pathogenic Th17 cells in EAE [18]. Moreover, IL-10 production by Th17 cells has been strongly related to the acquisition of regulatory properties by Th17 cells and the resolution of intestinal inflammation [20,21].

Extracellular ATP (eATP) is a danger signal released by dying and damaged cells, and it functions as an immunostimulatory signal that promotes inflammation [26,27]. eATP can be sensed by purinergic P2 receptors including the cation-selective receptor channels (P2X) and metabotropic G protein-coupled receptors (P2Y) [28]. eATP has been shown to engage P2 receptors on T cells to induce Th17 differentiation [29]. On the other hand, high doses of eATP induces necrotic lysis through P2X7 receptor signaling in T cells [30,31].

CD39 (NTPDase-1) and CD73 (ecto-5’-nucleotidase) ectonucleotidases are two cell-surface ectoenzymes that dephosphorylate ATP into its metabolites ADP, AMP and adenosine thus shifting the balance from inflammatory to suppressive microenvironments [32]. Recently, it has been reported that ATP signaling through P2X7 receptor inhibits the conversion of naive T cells to Tr1 cells and that CD39 can promote Tr1 differentiation through eATP hydrolysis [33].

It is have been described that Th17 cells generated with TGF-β1 and IL-6 express CD39 and CD73 ectonucleotidases endowing these cells with the capacity to produce adenosine and promote immunosuppressive microenvironments [34]. However, the role of CD39 expression in other aspects such as in depleting eATP to reduce P2X7 receptor-mediated cytotoxicity or promoting the plasticity of this subset of T cells has not been explored. In the current study, we present a comprehensive comparison of *in vitro*-generated Th17 cells expressing high and low levels of CD39 and CD73 ectonucleotidases. We found that Th17 cells generated with TGF-β1 (Th17_TGF-β1_) which express the CD39 and CD73 ectonucleotidases and Th17 cells generated with IL-23 (Th17_IL-23_) which do not express these ectonucleotidases constitute two fundamentally different Th17 subsets, evidenced at the transcriptional level and by the types of cytokines they secrete. In agreement with their transcriptional profile, Th17_TGF-β1_ cells were significantly less pathogenic than Th17_IL-23_ cells in a model of experimental colitis, as they induce a transient reduction in weight loss in Rag^-/-^ mice and the production of IL-10 in the intestine. Interestingly, Th17_TGF-β1_ cells can hydrolyze ATP in a CD39-dependent manner, providing Th17_TGF-β1_ cells a superior survival capacity when exposed to high levels of ATP. Furthermore, here we show that Th17_TGF-β1_ cells produce higher levels of IL-10 than Th17_IL-23_ cells when reactivated *in vitro* in the presence of Tr1-polarizing cytokines. Finally, we report that CD39 activity is important for IL-10 production by Th17_TGF-β1_ cells since CD39 inhibition using the specific inhibitor ARL67156 reduced IL-10 production by *in vitro* re-activated Th17 cells.

## Materials and Methods

### Mice

C57BL/6, B6SJL-PTPRC (CD45.1), OT-II, IL-17-GFP, Rag1^-/-^, P2X7R^-/-^ mice were purchased from The Jackson Laboratory. All mice were kept in an animal facility under standard housing guidelines. Animal work was carried out under institutional regulations of Fundación Ciencia & Vida and was approved locally by the ethical review committee of the Facultad de Ciencias, Universidad de Chile.

### Generation of Th17 cells

CD4+ T cells were purified from spleens of IL-17-GFP and P2X7R-/- mice. The spleen was perfused with RPMI + 10% FCS, and CD4+ T cells were positively selected using anti-CD4 MACS (Miltenyi Biotec) following the manufacturer’s instructions. CD4+ T cells were cultured in a 96-well flat bottom microplate (0.1 x 10^6^ CD4+ T cells/well) and were activated with plate-bound a-CD3 (2 μg/ml; clone 145-2C11, eBioscience) and a-CD28 (2 μg/ml; clone 37.51) for 4 days in the presence of different cytokine cocktails. To generate Th17_TGF-β1_ cells, CD4+ T cells were differentiated in the presence of 2 ng/ml recombinant human TGF-β1 (eBioscience), 20 ng/ml recombinant mouse IL-6 (eBioscience), 10 ng/ml IL-1β (eBioscience) and 5 μg/ml of anti-IFN-γ (clone XMG1.2, Biolegend) and then reactivated for another 3 days in the presence of 2 ng/ml recombinant human TGF-β1 (eBioscience) and 20 ng/ml recombinant mouse IL-6 (eBioscience). Th17_IL-23_ cells were differentiated in the presence of 2 ng/ml recombinant human TGF-β3 (eBioscience), 20 ng/ml recombinant mouse IL-6 (eBioscience), 10 ng/ml IL-1β (eBioscience) and 5 μg/ml of anti-IFN-γ (clone XMG1.2, Biolegend) and then reactivated in the presence of 20 ng/ml recombinant mouse IL-6 (eBioscience), 10 ng/ml IL-1β (eBioscience) and 25 ng/ml recombinant mouse IL-23 (Biolegend). Cells were then isolated by cell sorting for adoptive transfer experiments, RNA extraction, intracellular cytokine staining and flow cytometry.

### Induction of colitis in Rag^-/-^ mice

For experimental colitis experiments, 1.3x10^6^ Th17_TGF-β1_ or Th17_IL-23_ cells were sorted based on IL-17 production (GFP+) and then transferred into Rag^-/-^ mice. The body weight was measured every 2 days. Six weeks after adoptive transfer, the mice were sacrificed, and the entire colon was removed from cecum to anus. The colon length was measured as an indicator of inflammation. Clinical score was calculated based on weight loss and colon length. Weight-loss scores were determined as 0 = 0–2.5% weight loss; 1 = 2.5–5% weight loss; 2 = 5–7.5% weight loss; 3 = 7.5–10% weight loss; and 4 = >10% weight loss. This score was calculated using the weight of each mouse at the end point. Each weight data was compared to the average weight of control group. Colon length scores were determined as 0 = no colon size reduction; 1 = 0–5% colon size reduction; 2 = 5–10% colon size reduction; 3 = 10–15% colon size reduction; and 4 = >15% colon size reduction. This score was calculated using colon length normalized by the weight of each mouse. For each mouse, these scores were combined and divided by two to give an overall clinical score ranging from 0 (healthy) to 4 (maximal colitis).

### Analysis of transferred cells in Rag^-/-^ mice

Six to eight weeks after adoptive transfer of Th17_TGF-β1_ or Th17_IL-23_ cells into Rag-/- mice, the mice were sacrificed and lymphoid organs and lamina propria were dissected. The cells were analyzed by flow cytometry to assess the percentage of the transferred cells (CD3+ CD4+) within a lymphoid gate and the production of cytokines by intracellular cytokine staining.

### Intracellular staining and flow cytometry

Cells obtained from lamina propria, lymph nodes and *in vitro*-generated Th17 cells were re-stimulated with 0.25 μM PMA (Sigma-Aldrich) and 1 μg/ml ionomycin (Sigma-Aldrich) in the presence of GolgiPlug (BD Biosciences) for 4 h. Cells were stained with antibodies against the cell surface markers CD4, CD39, CD73, and then resuspended in a fixation/permeabilization solution (Cytofix/Cytoperm; BD Pharmingen). Following fixation and permeabilization, the cells were incubated with antibodies against IFN-γ, IL-17 and IL-10 for 30 min at 4°. The cells were then washed with permeabilization buffer and resuspended in PBS + 2% FCS for FACS analysis (FACSCanto II; BD Bioscience). In some cases, Fixable Viability Dye (eBioscience) was used to discard dead cells from the analysis. Analysis of FACS data was performed using the FLOWJO software (Tree Star Inc., Ashland, OR).

### Cytokine secretion measurements

Th17_TGF-β1_ or Th17_IL-23_ cells were activated for 3 h at 1x10^6^ cells/ml with 0.25 μM PMA (Sigma-Aldrich) and 1 μg/ml ionomycin (Sigma-Aldrich). After activation, the supernatants were harvested and analyzed using the mouse Th1/Th2/Th17 CBA Kit (BD Biosciences), following the manufacturer’s instructions. GM-CSF was analyzed by ELISA using the BD OptEIA kit (BD Biosciences, 555167).

To analyze the cytokines produced in the intestine of Rag^-/-^ mice transferred with Th17 cells, the intestine was cut into fragments of 1 cm of length and incubated in 1 ml IMDM + 10% FCS for 24 h at 37°C and 5% CO_2_. The medium was collected and centrifuged at 600 x g for 7 min. The supernatant was analyzed using the CBA Kit Mouse Th1/Th2/Th17 (BD Biosciences).

### qPCR

Th17 cells were isolated by cell sorting, and total RNA was obtained using EZNA Total RNA Kit I (Ω Bio-Tek). 1 μg of RNA was reverse transcribed using M-MLV reverse transcriptase (Invitrogen). The PCR reaction was performed using Brilliant II SYBR Green QPCR Master Mix (Agilent Technologies) in a Stratagene Mx3000P real-time PCR machine. For relative quantitation, the amplified fragments were normalized according to constitutive transcription of the housekeeping gene GAPDH. The sequences of the primers used for quantification of each measured transcript were the following:

*rorc* forward 5’-CAGAGGAAGTCAATGTGGGA-3’,

reverse 5’-GTGGTTGTTGGCATTGTAGG-3’;

*tbx21* forward 5’-CCTGTTGTGGTCCAAGTTCAAC-3’

reverse 5’-CACAAACATCCTGTAATGGCTTGT-3’;

*il17a forward 5’*TTCATCTGTGTCTCTGATGCT-3’

reverse 5’-AACGGTTGAGGTAGTCTGAG-3’;

il9 forward 5’-CTGATGATTGTACCACACCGTGC-3’

reverse 5’-GCCTTTGCATCTCTGTCTTCTGG-3’;

*il10 forward 5’*GAAGACAATAACTGCACCCA-3’

reverse 5’-CAACCCAAGTAACCCTTAAAGTC-3’;

*Il22 forward 5’*GACAGGTTCCAGCCCTACAT-*3’*

*reverse 5’*ATCGCCTTGATCTCTCCACT-*3’;*

*csf2 forward 5’*ACCACCTATGCGGATTTCAT-*3’*

*reverse 5’*TCATTACGCAGGCACAAAAG-*3’;*

*ifng forward 5’*GAGCCAGATTATCTCTTTCTACC-*3’*

*reverse 5’*GTTGTTGACCTCAAACTTGG-*3’;*

*grzb forward 5’*ATCAAGGATCAGCAGCCTGA-*3’*

*reverse 5’*TGATGTCATTGGAGAATGTCT-*3’;*

ahr forward 5’-CAGCAGATGCCTTGGTCTTCT-3’

reverse 5’-ATACGCTCTGATGGATGACATCA-3’;

cmaf forward 5’-AGCAGTTGGTGACCATGTCG-3’

reverse 5’-TGGAGATCTCCTGCTTGAGG-3’;

p2rx7 forward 5’-CCAGGAAGCAGGAGAGAACTT-3’

reverse 5’-ATCCGTGTTCTTGTCATCCAG-3’;

hprt forward 5’-CTCCTCAGACCGCTTTTTGC-3’

reverse 5’-TAACCTGGTTCATCATCGCTAATC-3’;

*gapdh forward 5’*TCCGTGTTCCTACCCCCAATG-*3’*

*reverse 5’*GAGTGGGAGTTGCTGTTGAAG-*3’*.

### Determination of ATP/AMP hydrolysis by HPLC

The enzymatic activity of CD39 and CD73 was evaluated based on the percentage of hydrolysis of ATP or AMP respectively by HPLC. Briefly, Th17 cells generated *in vitro* were diluted in Hanks’ balanced salt solution (HBSS) and incubated in a 96- well flat-bottom plates at 0.5x10^5^ cells/well with 10 μM ATP (Sigma-Aldrich) in the presence or absence of the CD39 inhibitor ARL67156 (Sigma-Aldrich) at a concentration of 50 μM or with 10 μM AMP (Sigma-Aldrich), with or without the CD73 inhibitor APCP (Adenosine 5’-(a,b-methylene) diphosphate) (50 μM) (Sigma-Aldrich). After 1 h, the cells were harvested, transferred to ice for 15 min, and then centrifuged at 1000 x g for 10 min. Supernatants were collected and stored at -20°C until further analysis. HPLC analysis was carried out in a Water Breeze system using an anion exchanger column (Mono Q; GE Healthcare, Chalfont St Giles, UK). The mobile phase used consisted of a linear gradient from buffer A (Tris–HCl 100 mM, pH 7.8) to buffer B (Tris–HCl 100 mM, NaCl 1 M, pH 7.8). The effluent was monitored at 257 nm using an online UV detector. The column was calibrated using ATP, AMP and adenosine as standards.

### Determination of Th17 cell survival in high concentrations of ATP

Th17 cells isolated by cell sorting were resuspended at 1x10^5^ cells/well in HBSS medium. Cells were cultured in the presence or absence of ATP (100, 500 or 1000 μM) (Sigma-Aldrich) for 30 min at 37°C and 5% CO_2_. Cells were then harvested and centrifuged at 600 x g for 7 min and resuspended in 100 μL of Binding Buffer (10 mM HEPES, 140 mM NaCl, 2.5 mM CaCl2, pH 7.4) containing 0.5 μL of Annexin V APC (Biolegend) and 2 μL of propidium iodide (50 μg/mL) (Sigma). Cells were incubated for 20 min at room temperature and 300 μL of Binding Buffer was added. Live and dead cells were analyzed by flow cytometry.

### Statistical analysis

Data are presented as mean ± SEM. Differences between groups were determined using Mann-Whitney test or two-tailed t-test. Where indicated, differences were analysed using Kruskal-Wallis or two-way analysis of variance paired with Bonferroni post-tests. Statistical analysis and graphs were obtained with GRAPHPAD PRISM (GraphPad Software Inc., La Jolla, CA).

## Results

### *In vitro*generated Th17 cells expressing high levels of CD39 ectonucleotidase present a regulatory phenotype

**Fig 1:**
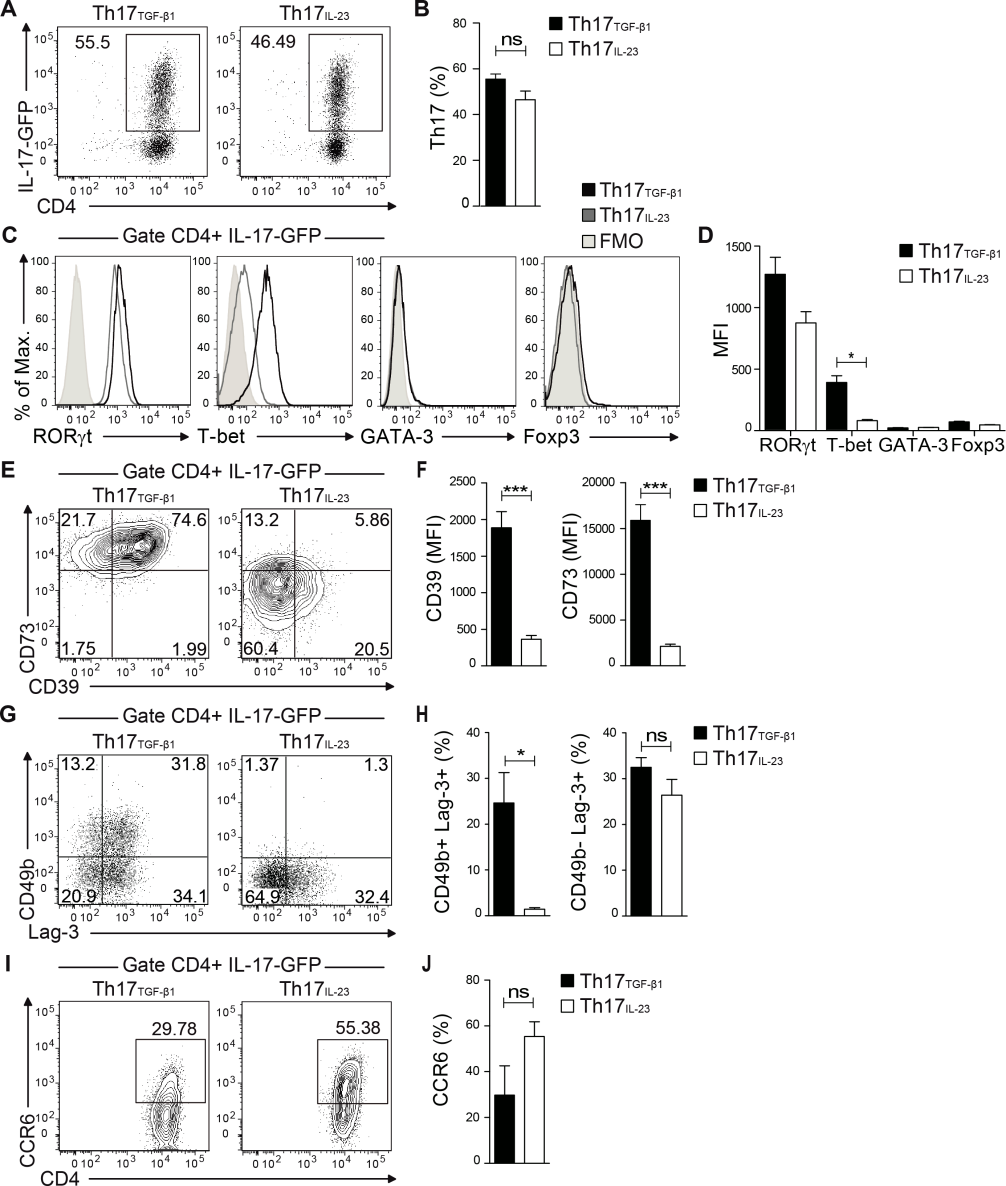
*In vitro*-generated Th17_TGF-β1_ but not Th17_IL-23_ cells express CD39 and CD73 ectonucleotidases. (A) IL-17-GFP expression in Th17 cells differentiated in the presence of TGF-β1 and IL-6 (Th17_TGF-β1_ cells) or IL-23, TGF-β3 and IL-1β (Th17_IL-23_ cells). (B) Percentage of IL-17-GFP+ cells among total CD4+ cells (n = 12). (C and D) FACS analysis and mean fluorescence intensity for RORγt, T-bet, GATA-3 and Foxp3 transcription factors in IL-17-GFP+ Th17 cells (n = 4). (E and F) Representative FACS analysis and mean fluorescence intensity for CD39 and CD73 ectonucleotidases (n = 7). (G) CD49b and Lag-3 (n = 4) expression in IL-17-GFP+ Th17 cells. (H) Percentage of CD49b+/Lag-3+ cells and percentage of CD49b-/Lag-3+ cells in IL-17-GFP+ Th17 cells (n = 4). (I and J) CCR6 expression in IL-17-GFP+ Th17 cells (n = 6). Data are presented as mean ± S.E.M. *p<0.05 and ***p<0.001 determined by t-test (B and F) or Mann-Whitney test (D, G and I).

To evaluate a putative role of CD39 and CD73 in Th17 cells we generated Th17 cells expressing high or low levels of these ectonucleotidases. It has been reported that TGF-β1 induces CD39 and CD73 expression in Th17 cells; however after a second round of restimulation in the absence of TGF-β1, Th17 cells lose ectonucleotidase expression [34]. For this reason, we generated Th17 cells using two rounds of activation. Th17 cells expressing high levels of CD39 and CD73 were generated with TGF-β1, IL-6, and IL-1β, and reactivated in the presence of TGF-β1 and IL-6 (Th17_TGF-β1_). Th17 cells expressing low levels of ectonucleotidases were generated with TGF-β3, IL-6, and IL-1β, and reactivated in the presence of IL-6, IL-1β, and IL-23 (Th17_IL-23_). As shown in Fig 1A and 1B, a similar percentage of IL-17 producing cells were obtained in Th17_TGF-β1_ and Th17_IL-23_ culturing conditions. Accordingly, Th17_TGF-β1_ and Th17_IL-23_ cells expressed similar levels of RORγt, the master transcription factor of Th17 cells and neither expressed GATA-3 nor Foxp3 transcription factors. Interestingly, although it has been reported that Th17 cells generated with IL-23 express higher levels of T-bet than cells generated with TGF-β1 [24,35], in our setting Th17_TGF-β1_ cells expressed higher levels of this master transcription factor compared to Th17_IL-23_ cells (Fig 1C and 1D).

Next, we analyzed CD39 and CD73 expression by these *in vitro*-generated Th17 cells. As expected, Th17_TGF-β1_ cells acquired high levels of CD39 and CD73 ectonucleotidases expression whereas Th17_IL-23_ cells presented low expression of both ectonucleotidases (Fig 1E and 1F). A fraction of Th17_TGF-β1_ cells (approx. 25%) also expressed significant levels of CD49b and Lag-3, markers of Tr1 cells [36], whereas a fraction of Th17_IL-23_ cells only expressed Lag-3. Although a fraction of Th17_TGF-β1_ cells showed Tr1 markers, these do not constitute *bona fide* Tr1 cells, since they express RORγt transcription factor and produce IL-17. Although both Th17 cell subsets expressed CCR6, Th17_TGF-β1_ cells showed lower levels of this chemokine receptor compared to Th17_IL-23_ cells (Fig 1G and 1J).

**Fig 2:**
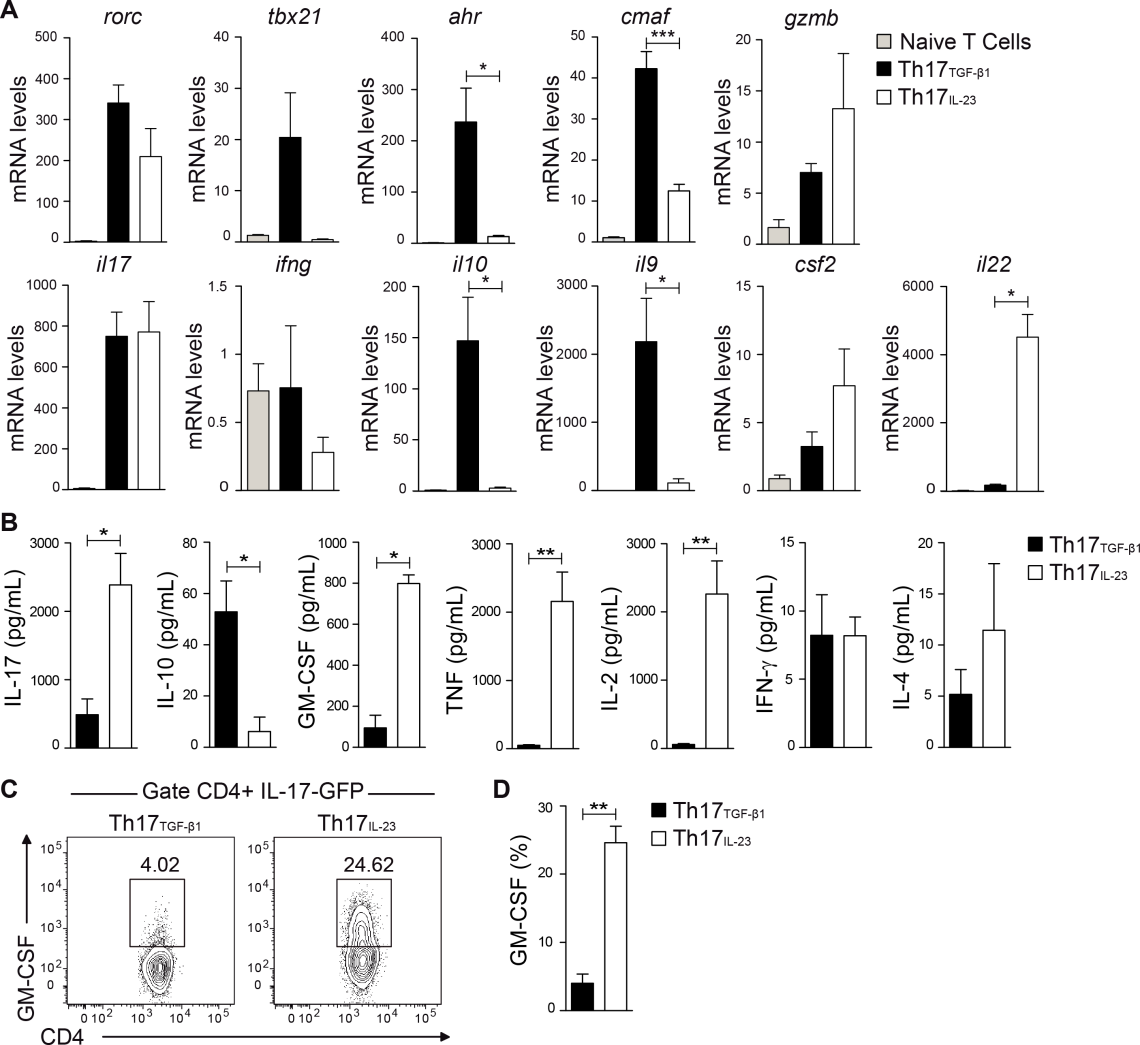
Th17_TGF-β1_ cells present a regulatory phenotype. (A) IL-17-GFP+ Th17_TGF-β1_ and Th17_IL-23_ cells were sorted and then analyzed by real-time PCR for mRNA expression of several transcription factors and cytokines (n = 3). (B) IL-17-GFP+ Th17_TGF-β1_ and Th17_IL-23_ cells were sorted and then reactivated for 4 hrs with PMA plus ionomycin to assess cytokine production by CBA or (C) in the presence of PMA, ionomycin and brefeldin A to analyze GM-CSF production by FACS (n = 5). (D) Percentage of GM-CSF+ cells within IL-17-GFP+ Th17_TGF-β1_ and Th17_IL-23_ cells (n = 5). Data are presented as mean ± S.E.M. *p<0.05, **p<0.01 and ***p<0.001 determined by t-test (A) or Mann-Whitney test (B and D).

We further investigated the expression of Th17-associated genes in Th17_TGF-β1_ and Th17_IL-23_ cells. The expression of mRNAs encoding T-bet, IFN-γ, IL-9, AHR, c-Maf and IL-10 was higher in Th17_TGF-β1_ cells whereas expression of mRNAs encoding GM-CSF and IL-22 was higher in Th17_IL-23_ cells (Fig 2A). Interestingly, following *in vitro* reactivation, Th17_TGF-β1_ cells secreted higher levels of IL-10, and lower levels of IL-17, GM-CSF, TNF, IL-2 compared to Th17_IL-23_ cells (Fig 2B–2D). Taken together, these results strongly suggest that Th17_TGF-β1_ cells rapidly lose their inflammatory potential and present a regulatory phenotype upon reactivation.

### *In vitro*generated Th17 cells expressing high levels of CD39 and CD73 can hydrolyze ATP and survive to ATP-induced death

**Fig 3:**
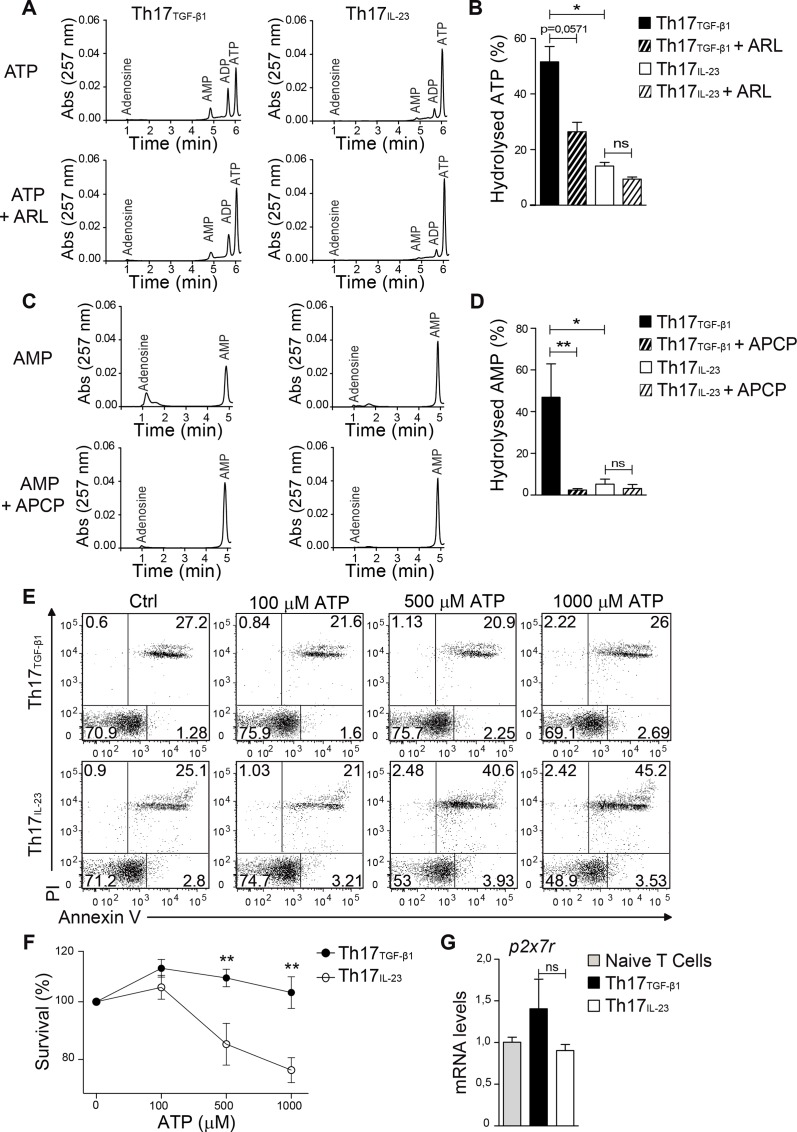
Th17_TGF-β1_ but not Th17_IL-23_ cells hydrolyze ATP to adenosine in a CD39-and CD73-dependent manner and survive in the presence of high doses of ATP. IL-17-GFP+ Th17_TGF-β1_ and Th17_IL-23_ cells were sorted and cultured for 1 hr with 10 μM ATP in the presence of 50 μM ARL67156 or 50 μM APCP. Supernatants were then analyzed by HPLC to assess (A and B) ATP and (C and D) AMP hydrolysis (n = 5). (E) Representative FACS analysis of Th17 cell survival (Annexin V-/PI-) in the presence of graded doses of ATP. (F) Percentage of Th17 cell survival in the presence of ATP (n = 3). (G) IL-17-GFP+ Th17_TGF-β1_ and Th17_IL-23_ cells were sorted and then analyzed by real-time PCR to assess mRNA encoding P2X7 receptor (n = 4). Data are presented as mean ± S.E.M. *p<0.05 and **p<0.01 determined by Mann-Whitney test (B and D), two-way analysis of variance (F) or t-test (G).

We next tested the enzymatic activity of CD39 and CD73 ectonucleotidases in Th17 cells. As shown in Fig 3A–3D, only Th17_TGF-β1_ cells, which express the CD39 and CD73 ectonucleotidases, can hydrolyze ATP and AMP. Moreover, ATP hydrolysis was partially blocked in Th17_TGF-β1_ cells by inhibiting CD39 enzymatic activity using the ecto-ATPase inhibitor ARL67156 while adenosine production was blocked using the CD73 inhibitor APCP in these cells.

As Th17_TGF-β1_ cells express the CD39 and CD73 ectonucleotidases and produce adenosine, we tested whether these cells present suppressive capacity *in vitro*. For this, Th17_TGF-β1_ or Th17_IL-23_ cells were sorted based on IL-17-GFP expression and co-cultured with Violet-labeled CD4+ effector T cells from OT-II mice activated with OVA_323-339_ and antigen presenting cells for 3 days. As shown in S1 Fig, both Th17_TGF-β1_ and Th17_IL-23_ cells displayed a low suppressive capacity only when co-cultured in direct contact with effector cells at high Th17/Teff ratios (1:1). No suppression was observed when the cells were cultured using transwell chambers (S1 Fig), suggesting that soluble factors such as adenosine are not involved in this process.

Since Th17_TGF-β1_ cells can hydrolyze ATP, we sought to determine whether these cells were more resistant to ATP-induced cell death. As shown in Fig 3E and 3F, Th17_TGF-β1_ cells were more resistant than Th17_IL-23_ cells to rapid cell death induced by high doses of ATP (>500μM). Since both types of Th17 cells express similar levels of the mRNA encoding the P2X7 receptor (Fig 3G), this suggests that ATP signaling through the P2X7 receptor is reduced in Th17_TGF-β1_ cells due to CD39 activity. These results also raise the possibility that CD39 expression confers Th17_TGF-β1_ cells with a superior survival capacity when faced with toxic doses of ATP.

### *In vitro*generated Th17 cells expressing high levels of CD39 ectonucleotidase convert to IL-10 producing cells during intestinal inflammation

**Fig 4:**
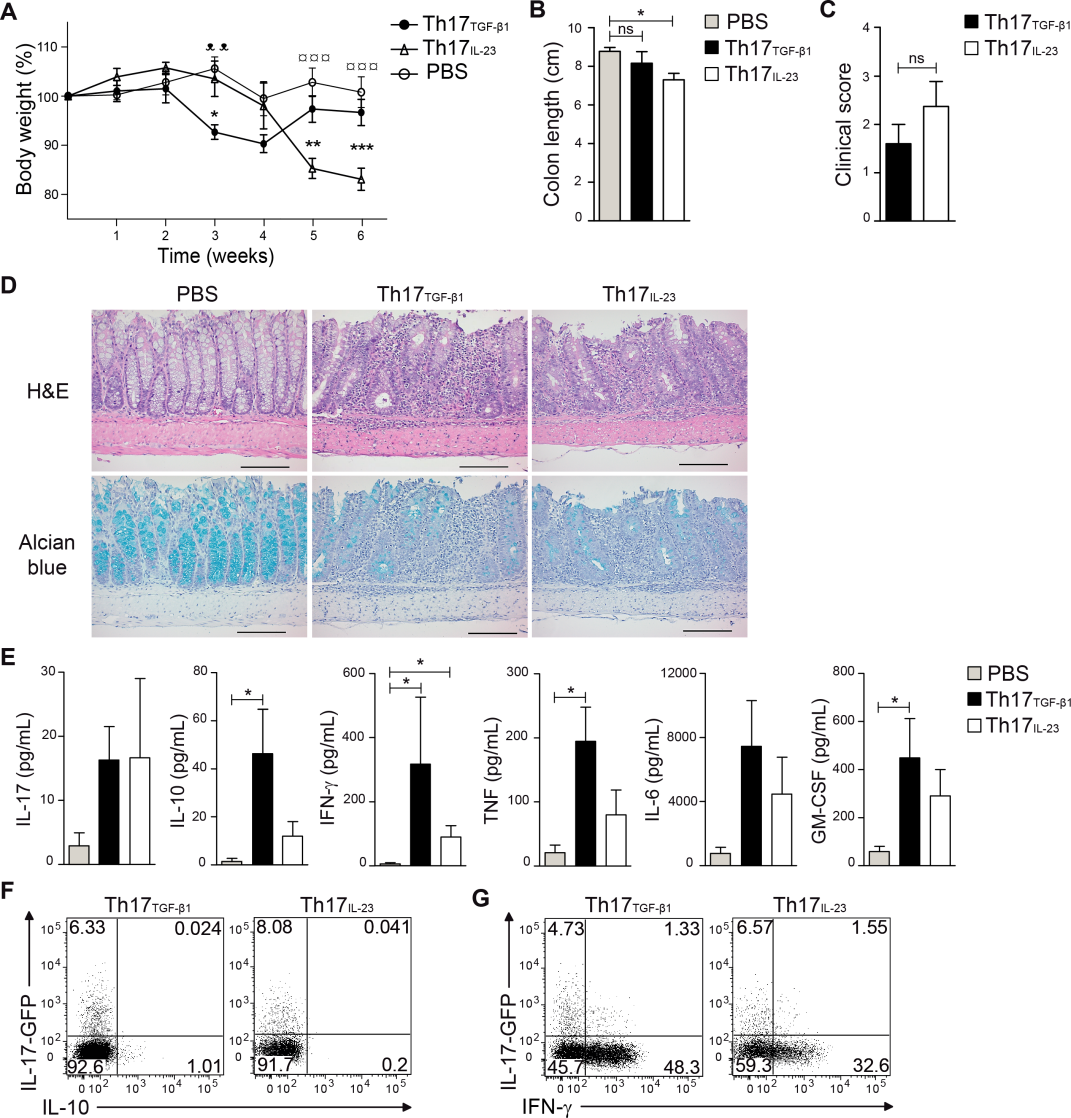
Th17_TGF-β1_ cells are less colitogenic than Th17_IL-23_ cells and produce IL-10 and IFN-γ *in vivo*. 1.3x10^6^ IL-17-GFP+ Th17_TGF-β1_ and Th17_IL-23_ cells were transferred to Rag1^-/-^ mice. (A) The weight of mice was measured over the course of 6 weeks after adoptive transfer of Th17 cells (n = 5–8 mice per group). (B) Colon length was measured 6 weeks following transfer of Th17 cells (n = 5). (C) Clinical score was calculated based on weight loss and colon length 6 weeks after adoptive transfer of Th17 cells (n = 5). (D) Colonic histopathology. H&E and alcian blue staining, original magnification 20X. Scale bar 100 μm (E) To determine intestinal cytokine production, intestinal tissues of Th17 recipient mice were cultured for 24 hs at 37°C and 5% CO_2_ and production of several cytokines was analyzed by CBA (n = 6). (F and G) Representative FACS analysis of IL-17, IL-10 and IFN-γ production by Th17_TGF-β1_ and Th17_IL-23_ cells 6 weeks after adoptive transfer to Rag1^-/-^ mice. Data are presented as mean ± S.E.M. *p<0,05, **p<0,01 and ***p<0,001 comparing Th17_TGF-β1_ and Th17_IL-23_; ᵜᵜp<0,01 comparing PBS and Th17_TGF-β1_; ^¤¤¤^p<0,001 comparing PBS and Th17_IL-23_ determined by two-way analysis of variance (A). *p<0,05 determined by Kruskal-Wallis test (B,C and E).

It has been reported that Th17 cells generated with TGF-β1 and IL-6 are less pathogenic than Th17 cells differentiated with IL-23 [18]. To evaluate the pathogenicity of our Th17 cells in a model of experimental colitis, we transferred Th17_TGF-β1_ or Th17_IL-23_ cells into Rag^-/-^ mice and measured body weight loss and colon length in these mice. As shown in Fig 4A, mice treated with Th17_TGF-β1_ cells started losing body weight 3 weeks after adoptive transfer whereas mice transferred with Th17_IL-23_ cells presented a delay in the onset of weight loss and started losing weight 5 weeks following adoptive transfer. Moreover, Rag^-/-^ mice treated with Th17_TGF-β1_ cells presented a transient weight loss and began to recover by week 5, whereas mice treated with Th17_IL-23_ cells presented severe weight loss and had to be sacrificed by 6 weeks after the adoptive transfer (Fig 4A). In agreement with the severe and persistent weight loss, mice treated with Th17_IL-23_ cells presented a significant reduction in the colon length compared to mice receiving PBS (Fig 4B). Although not statistically significant, mice treated with Th17_TGF-β1_ cells presented a lower clinical score compared to mice treated with Th17_IL-23_ cells (Fig 4C). H&E and alcian blue staining of distal colonic sections revealed severe wall thickening, extensive leukocyte infiltration, and disruption of intestinal crypts and goblet cells in mice treated with Th17_TGF-β1_ or Th17_IL-23_ cells compared to controls (Fig 4D). This suggests that although mice treated with Th17_TGF-β1_ cells recover and gain weight, the damage to the colon is not reversed in these mice at this time point.

In agreement with the less severe body weight loss, mice treated with Th17_TGF-β1_ cells secreted higher levels of IL-10 as determined directly in their small intestine compared to mice treated with Th17_IL-23_ cells (Fig 4E). We next analyzed the stability of Th17_TGF-β1_ and Th17_IL-23_ cells 6 weeks after adoptive transfer and found that both Th17 cell subsets lost IL-17 production and a significant fraction of transferred cells produced IFN-γ (Fig 4G). Notably, a small fraction of transferred Th17_TGF-β1_ cells, but not of Th17_IL-23_ cells, were able to produce IL-10 (Fig 4F). The differences observed in body weight loss were not due to a differential capacity of of Th17_TGF-β1_ and Th17_IL-23_ cell subsets to survive or persist in the host, since both populations were found at similar percentages within spleen, mesenteric lymph nodes and small intestine lamina propria up to 8 weeks following adoptive transfer into Rag^-/-^ mice (S2 Fig). Taken together, these data suggest that Th17_TGF-β1_ cells are able to induce the production of IL-10 in the intestine of Rag^-/-^ mice and can convert into IL-10-producing cells during the recovery from intestinal inflammation, which may explain the transient weight loss observed in mice treated with Th17_TGF-β1_ cells.

### CD39-mediated ATP hydrolysis is crucial for *in vitro* Th17 conversion to IL-10 producing cells

**Fig 5:**
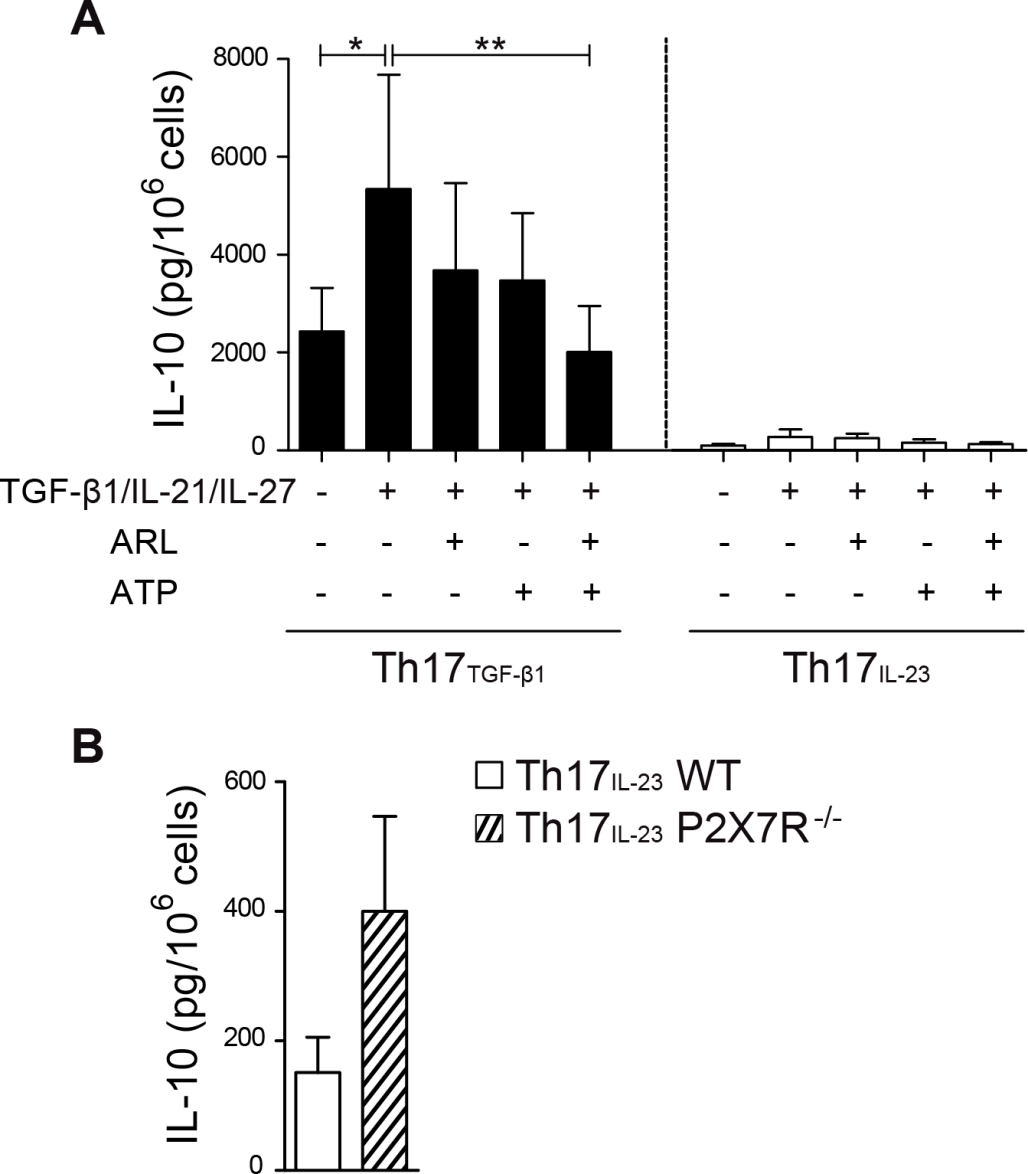
ATP hydrolysis by CD39 on Th17_TGF-β1_ cells promotes their conversion into IL-10-producing cells. (A) IL-10 production by IL-17-GFP+ Th17_TGF-β1_ and Th17_IL-23_ cells restimulated for 3 days with anti-CD3 and anti-CD28 antibodies in the presence and absence of Tr1 polarizing cytokines (TGF-β1, IL-21 and IL-27), 50 μM ATP and 250 μM ARL67156. IL-10 production was analyzed by CBA (n = 4). (B) Th17_IL-23_ cells from wild-type and P2X7R knockout mice were restimulated for 3 days with anti-CD3 and anti-CD28 antibodies and IL-10 production was analyzed by CBA (n = 3). Data are presented as mean ± S.E.M. *p<0.05; **p<0.01 determined by repeated measures analysis of variance.

To test the possibility that Th17_TGF-β1_ cells differentiate into IL-10-producing cells more efficiently than Th17_IL-23_ cells, we reactivated IL-17 (GFP+) Th17 cells with anti-CD3 and anti-CD28 antibodies for 3 days and evaluated IL-10 production *in vitro*. As shown in Fig 5A, Th17_TGF-β1_ cells produced higher levels of IL-10 than Th17_IL-23_ cells following activation. Moreover, the addition of TGF-β1, IL-21, and IL-27 (an improved cytokine cocktail to stimulate IL-10 production and Tr1 cell differentiation) during the reactivation increased the levels of IL-10 production by Th17_TGF-β1_ cells.

Since it has been described that ATP inhibits naive T cell conversion to Tr1 cells via P2X7 receptor signaling [33], we tested whether CD39 and ATP hydrolysis is necessary for the conversion of Th17 cells to Tr1 cells. For this, both subsets of Th17 cells were tested in their ability to convert to IL-10-producing cells in the presence of Tr1 polarizing cytokines (TGF-β1/IL-21/IL-27), ATP and the CD39 specific inhibitor, ARL67156. As shown in Fig 5A, the addition of ATP reduced the ability of Th17_TGF-β1_ cells to produce IL-10 in the presence of ARL67156, demonstrating that CD39 enzymatic activity is important for IL-10 production. Interestingly, Th17_IL-23_ cells generated from P2X7 receptor knockout mice, produced higher levels of IL-10 when compared to Th17_IL-23_ cells generated from wild-type mice (Fig 5B). These results suggest that CD39 enzymatic activity is crucial for limiting P2X7 receptor signaling in Th17 cells, promoting the production of IL-10 and the conversion of Th17 cells into Tr1-like cells.

## Discussion

Extracellular ATP (eATP) is a danger signal released by dying and damaged cells, and it functions as an immunostimulatory signal that promotes inflammation [26,27]. CD39 and CD73 are two cell-surface ectoenzymes that dephosphorylate eATP into its metabolites, ADP, AMP, and adenosine, in a tightly regulated process. CD39 catalyzes the conversion of eATP into AMP, whereas CD73, catalyzes the dephosphorylation of AMP into adenosine [37–39]. The coordinated action of these ectonucleotidases results in the generation of extracellular adenosine, a molecule known for its immunosuppressive properties [40].

It has been described that the addition of TGF-β and IL-6 during the *in vitro* differentiation of Th17 cells induces the expression of CD39 and CD73 ectonucleotidases by Th17 cells [34] and that tumor-infiltrating Th17 cells may express these ectonucleotidases. Although the expression of CD39 and CD73 ectonucleotidases has been related to their potential to generate adenosine and create an immunosuppressive microenvironment [34], they may also serve to deplete eATP. In this line of evidence, Falk and colleagues have reported that CD39 expression by Foxp3+ regulatory T cells (Tregs) is involved in eATP depletion and reduces the cytotoxic effects of this molecule [41]. Thus, CD39 expression may endow Tregs with the capacity to enter into inflamed sites and mediate immunosuppression by preventing P2X7 receptor-mediated cell death. Our results demonstrate that this mechanism may also be active on Th17 cells, where ATP-induced cell death is reduced in the population of Th17 cells (Th17_TGF-β1)_ expressing the CD39 ectonucleotidase. This result puts forward the idea that Th17 cells expressing CD39 may survive in ATP-rich sites such as an inflamed tissue.

Th17 cells have been defined as a plastic subset of T cells, being able to differentiate into several other T cell population during inflammation [42]. We and others have reported that Th17 cells can differentiate into IFN-γ-producing cells when transferred into lymphopenic hosts, during EAE or in a murine melanoma model [11,13–15]. Moreover, Flavell and colleagues have shown that Th17 cells are also able to differentiate into IL-10-producing Tr1 cells during the resolution of inflammation and that this population of Tr1 cells present regulatory properties as they abolished Th17 cell-mediated colitis [20,21]. In this study, we confirmed that Th17 cells generated with TGF-β1 are a highly plastic subset of T helper cells, as they can differentiate into IFN-γ-and IL-10-producing cells following transfer into Rag^-/-^ mice.

Tr1 cells constitute an important subset of CD4+ T cells that help to control excessive inflammatory responses mainly through the production of IL-10 [43]. It has been reported that AHR and c-Maf transcription factors physically interact enabling the transactivation of the IL-10 promoter and thus are involved the differentiation of Tr1 cells [44,45]. Importantly, eATP and hypoxia have been shown to suppress the generation of Tr1 cells by triggering AHR inactivation through HIF1-α [46]. It has been demonstrated that eATP increases the interaction of HIF1-α to ARNT decreasing AHR binding to ARNT [33], resulting in the reduction of the transcription of AHR controlled genes. Interestingly, the group of Quintana reported that CD39 expression is important for the production of IL-10 by Tr1 cells as it allowed the depletion of eATP favoring AHR/ARNT interaction [33]. Based on this evidence, we tested whether CD39 may also play a role in promoting Th17 cell differentiation into IL-10-producing cells. In agreement with this hypothesis, Th17 cells expressing the CD39 ectonucleotidase (Th17_TGF-β1_) induce an IL-10-rich microenvironment when transferred in a setting of intestinal inflammation. Furthermore, Th17_TGF-β1_ cells produce higher levels of IL-10 compared to Th17_IL-23_ cells when re-activated *in vitro*. Importantly, in the presence of eATP and the CD39 inhibitor ARL67156 Th17_TGF-β1_ cells reduced their ability to produce IL-10.

Although we demonstrated adenosine production by Th17_TGF-β1_ cells, we could not detect a strong suppressive activity of Th17 cells over effector T cells, which could be due to the strong activation stimulus we used to induce effector T cell activation. These results are different from those reported by the group of Ghriringelli who suggest that adenosine produced by Th17 cells have a suppressor activity [34]. Our data strongly argue in favor of the idea that CD39 may not only be involved in generating a suppressive microenvironment, but also may deplete eATP allowing these cells to survive into inflamed tissues. On the other hand, CD39 expression may be a determining factor in the differentiation of Th17 cells to Tr1-like cells.

## Supporting Information

S1 Fig Th17 cells delay effector T cell proliferation in a contact-dependent manner. Proliferation of effector CD4+ T cells during *in vitro* suppression assays with Th17_TGF-β1_ or Th17_IL-23_ cells. Th17_TGF-β1_ or Th17_IL-23_ cells were sorted based on IL-17-GFP expression and co-cultured for 3 days at different ratios with Violet-labeled CD4+ effector T cells from OT-II mice activated with OVA_323-339_ and antigen presenting cells. (n = 3).
(TIF)

S2 Fig Th17_TGF-β1_ and Th17_IL-23_ subsets present similar *in vivo* persistence. 1.3x10^6^ IL-17-GFP+ Th17_TGF-β1_ and Th17_IL-23_ cells were transferred to Rag1-/- mice and the percentage of CD4+ CD3+ was analyzed 8 weeks after adoptive transfer in the spleen (A), mesenteric lymph node (B) and small intestine lamina propria (C) (n = 6–7 mice per group). Data are presented as mean ± S.E.M.
(TIF)
