# Supplementary figures and images for: Purinergic Signaling as a Regulator of Th17 Cell Plasticity
Source: PLoS One. 2016 Jun 20;11(6):e0157889. doi: 10.1371/journal.pone.0157889 (PMC4913941; doi:10.1371/journal.pone.0157889)

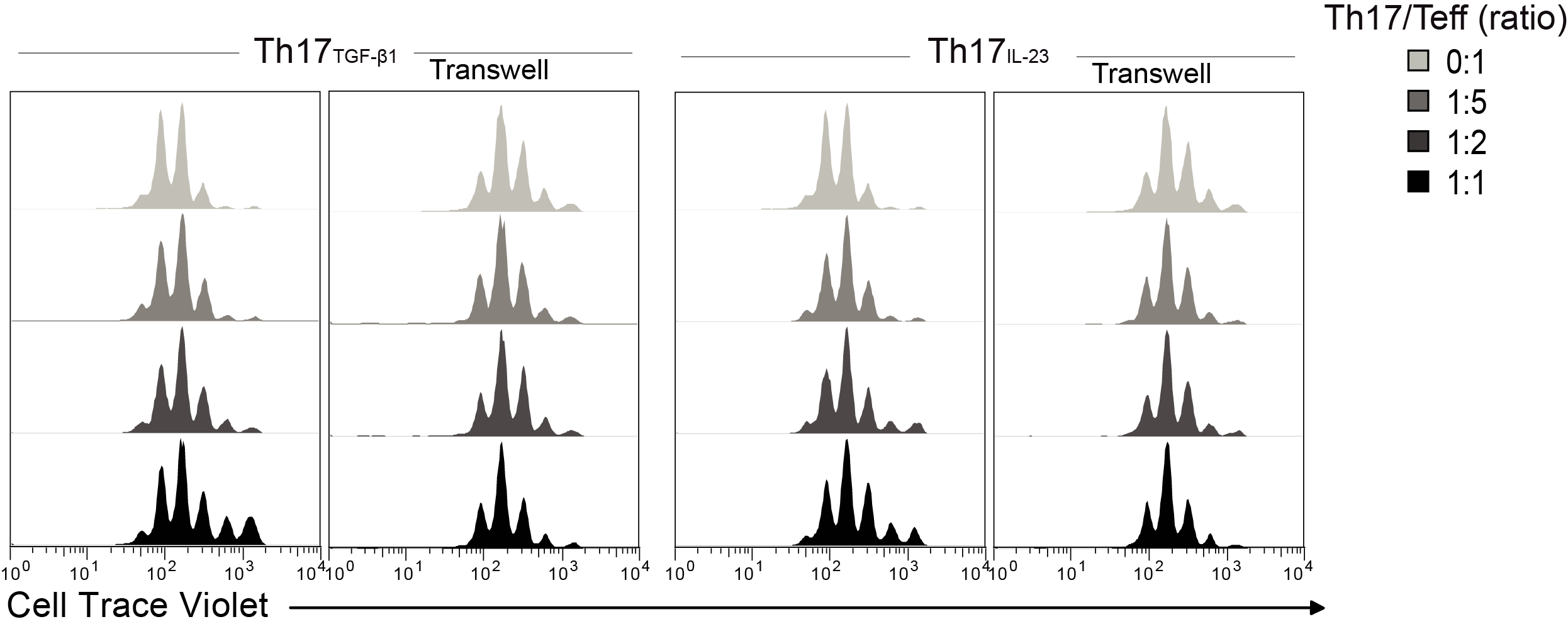

Supplement: S1 Fig — Proliferation of effector CD4+ T cells during in vitro suppression assays with Th17TGF-β1 or Th17IL-23 cells. Th17TGF-β1 or Th17IL-23 cells were sorted based on IL-17-GFP expression and co-cultured for 3 days at different ratios with Violet-labeled CD4+ effector T cells from OT-II mice activated with OVA323-339 and antigen presenting cells. (n = 3). (TIF) [file pone.0157889.s001.tif]

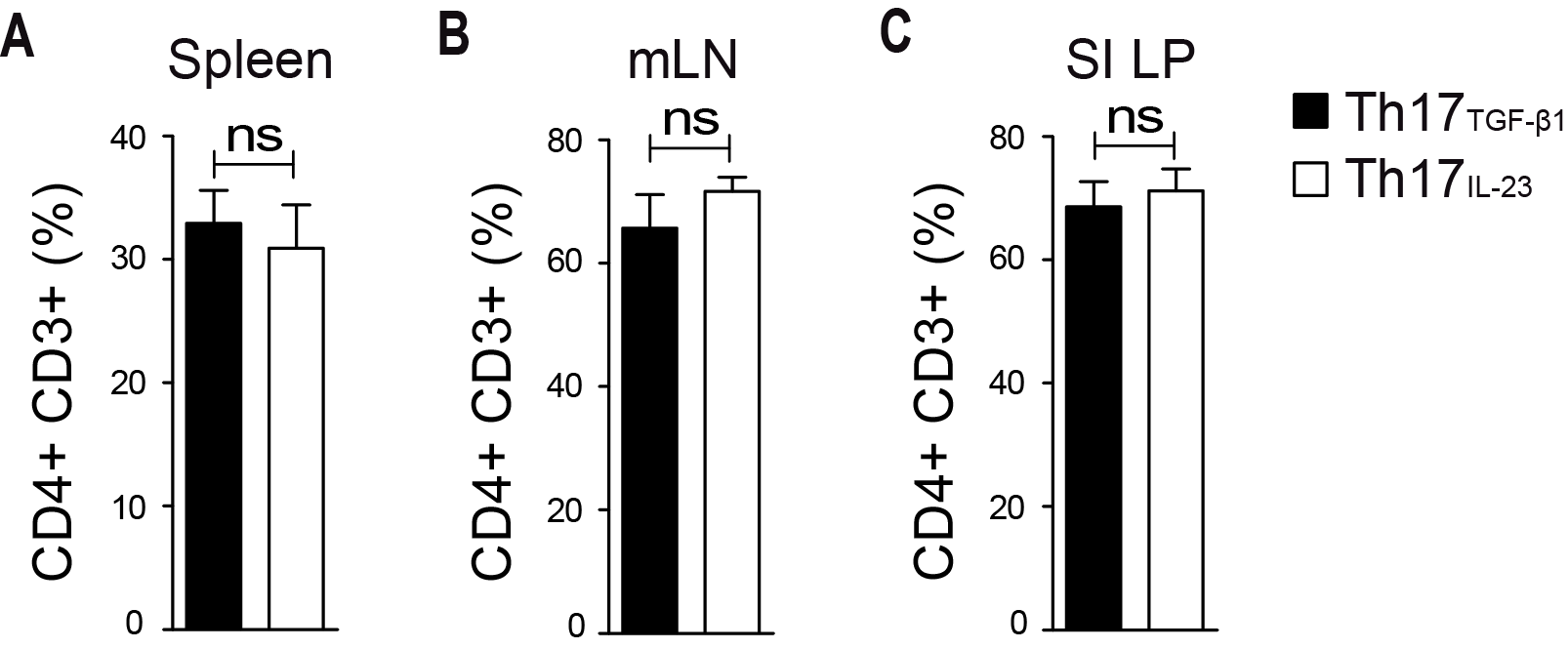

Supplement: S2 Fig — 1.3x10^6^ IL-17-GFP+ Th17TGF-β1 and Th17IL-23 cells were transferred to Rag1-/- mice and the percentage of CD4+ CD3+ was analyzed 8 weeks after adoptive transfer in the spleen (A), mesenteric lymph node (B) and small intestine lamina propria (C) (n = 6–7 mice per group). Data are presented as mean ± S.E.M. (TIF) [file pone.0157889.s002.tif]
